# EFFECT OF HOME-BASED REHABILITATION IN FRAIL OLDER ADULTS AFTER CARDIAC SURGERY: A PROSPECTIVE FEASIBILITY STUDY

**DOI:** 10.2340/jrm.v58.45667

**Published:** 2026-06-04

**Authors:** Ambre KOMONSKI, Killian JOUANNEAU, Anne Sophie BOUREAU, Jérémie HUET

**Affiliations:** 1 Nantes Université, CHU Nantes, Pôle de kinésithérapie, Nantes; 2 IFM3R, Saint Sébastien sur Loire; 3 Nantes Université, CHU Nantes, Pôle de Gérontologie Clinique, Nantes; 4 Nantes Université, CHU Nantes, CNRS, INSERM, l’Institut du Thorax, Nantes; 5 Nantes Université, CHU Nantes, Movement – Interactions – Performance, MIP, UR 4334, Nantes, France

**Keywords:** cardiac surgery, feasibility, frailty, rehabilitation

## Abstract

**Introduction:**

Frail older adults undergoing cardiac surgery are vulnerable to postoperative complications and functional decline. Vivifrail is a structured, multicomponent exercise programme designed to enhance physical performance. This study evaluated its feasibility in the postoperative period.

**Methods:**

A non-interventional, prospective, single-centre, study was conducted at Nantes University Hospital between March and December 2024. Patients aged ≥ 75 years, with a baseline Short Physical Performance Battery test (SPPB) score < 9 were included. SPPB, Timed Up and Go (TUG), and handgrip tests were performed at baseline (D0) and 6 weeks (W6). The primary outcome was to assess feasibility based on recruitment, retention rate, and physical performance at W6.

**Results:**

Among 45 eligible patients, 38 accepted the programme; 29 returned for assessment at W6. Mean programme completion rate was 54%. All functional assessments improved meaningfully over time, but adherence did not significantly modify these trajectories. However, adherent participants showed lower frailty at W6.

**Conclusion:**

Home-based rehabilitation using Vivifrail proved to be feasible for frail older adults during postoperative recovery. Clinically meaningful improvements were observed in physical performance during the first 6 weeks of the programme. Future research should confirm this trend and consider Vivifrail for patients who cannot access specialized rehabilitation centres.

## LAY ABSTRACT

Frail older adults often have a harder time recovering after heart surgery. They may lose strength, balance, and independence in the weeks following discharge from hospital. Exercise programmes can help, but many patients cannot attend specialized rehabilitation centres. We looked at whether a simple home-based exercise program called Vivifrail could be used safely and realistically by older adults after cardiac surgery. The programme includes strength, balance, and mobility exercises designed for frail individuals. We followed patients aged 75 years and older who started the programme after surgery at Nantes University Hospital. We measured their mobility, balance, and strength at the start and again 6 weeks later. Most patients agreed to try the programme, and more than half of them completed it. Overall physical performance improved during the 6 weeks. These results suggest that a home-based programme like Vivifrail may help frail older adults recover after heart surgery, especially when access to rehabilitation centres is limited.

The management of older patients represents a major public health concern, particularly after cardiac surgery, where acute stress may disrupt homeostasis and unmask a state of frailty, leading to functional decline (1). Frailty increases the risk of both fatal and non-fatal cardiovascular disease, and vice versa. The combination of these 2 factors also increases the risk of functional decline by a factor of 2 to 3 (2, 3). Given that frailty can be reversible over time (4, 5), frail older adults represent a key target population for interventions aimed at preventing disability (6, 7).

Cardiac rehabilitation (CR), including multicomponent interventions such as resistance, balance, flexibility exercises and gait retraining, has proven effective in maintaining functional independence (6, 8, 9). However, CR remains underutilized (10, 11), particularly among frail older adults (12). The absence of direct referral to rehabilitation upon hospital discharge is a major reason why most post-surgical patients fail to complete a CR programme (13). Both the limited diversity and specificity of prescribed exercises (14) and the insufficient information provided to patients (15) may also impair adherence.

These challenges were particularly evident at Nantes University Hospital, where, as early as 2020, geriatricians and cardiologists began questioning the limits of post-surgical follow-up care for older patients. Numerous reports indicated that around 70% of patients received no physiotherapy follow-up and felt insufficiently informed about the risks of undernutrition or psychological disorders after surgery (16).

Additional barriers to CR participation include geographical and logistical constraints such as long distances to rehabilitation centres (13, 15). To overcome these barriers, home-based interventions have been developed and shown to be as safe and effective as traditional CR in improving clinical outcomes and quality of life (17, 18).

As part of the development of a follow-up pathway for frail older adults after cardiac surgery at Nantes University Hospital, we selected the Vivifrail programme, recommended within the WHO’s ICOPE framework (19). This programme provides tailored physical exercises adapted to the functional level and fall risk of older adults. Vivifrail has demonstrated significant improvements in physical performance, and may also positively influence cognition, muscle function, and mood in frail populations (20–22).

Although Vivifrail has shown promising results in managing frailty, its implementation among postoperative cardiac patients has not yet been evaluated. This study aimed to assess the feasibility of the Vivifrail programme for frail older adults following cardiac surgery.

## MATERIALS AND METHODS

A non-interventional, prospective, single-centre study was conducted at Nantes University Hospital. The results were reported in accordance with STROBE guidelines. A published study protocol is available on OSF (https://osf.io/js7n4/?view_only=d122ebba23a94cf5b385e3ef1d19c246) and was approved by the Nantes Health Ethics Group (“Groupe Nantais d’Ethique dans le Domaine de la Santé”), Nantes, France (N°24-22-02-273).

### Participants

Participants were recruited from the Department of Thoracic and Cardiovascular surgery at Nantes University Hospital between March and December 2024. The target sample size was determined pragmatically according to the department’s recruitment capacity and logistical constraints. Two trained research physiotherapists (AK, ET) conducted the screening interviews.

Inclusion criteria were: age ≥ 75 years, recent cardiac surgery at Nantes University Hospital, a Short Physical Performance Battery (SPPB) score < 9 (out of 12) on hospital discharge, and provision of written informed consent.

Non-inclusion criteria included any medical condition preventing participation in the Vivifrail programme (i.e., persistent uncontrolled arrhythmia, hypertension or orthostatic hypotension after surgery, acute endocarditis or pericarditis, acute thromboembolic disease) and legal guardianship or curatorship status.

### Intervention

Within the 5 first days after surgery, a baseline assessment (D0) was conducted, including the SPPB, Timed Up and Go (TUG) test, and handgrip strength measurement. The SPPB combines balance, walking speed, and sit-to-stand (STS) tests into a composite score from 0 (worst) to 12 (best). A score ≤ 8 points indicates frailty (23, 24). The TUG performance was considered as a predictor of fall risk, with times ≥ 13.5 s indicating elevated fall risk (25, 26). These assessments were repeated at 6 weeks (W6) during a follow-up consultation. Patients also recorded daily exercise completion in a Vivifrail logbook, allowing the calculation of their adherence rate.

Participants were assigned to a specific Vivifrail programme level according to their physical functional status (SPPB score) and fall risk (recent falls, TUG, walking speed, mild cognitive impairment) (27):

- A = Severe limitation/dependent or disabled (SPPB 0–3);
- B = Moderate limitation/frail, fall risk (SPPB 4–6);
- B+ = Moderate limitation/frail, no fall risk (SPPB 4–6);
- C = Mild limitation/prefrail, fall risk (SPPB 7–9);
- C+ = Mild limitation/prefrail, no fall risk (SPPB 7–9);
- D = Minimal limitation/autonomous (SPPB 10–12).

Each patient received an exercise booklet corresponding to their level. The intervention consisted of a 6-week multi-component exercise programme performed 5 days per week. In addition to daily walking, participants at risk of falls performed specific resistance, balance, and stretching exercises 4 days per week, whereas others performed these exercises 3 days per week. All exercises from the Vivifrail programme were compatible with standard sternal precautions. Only one adaptation was required: “squeezing a ball” was used in place of “wringing out a towel”, in anticipation of contraindications to performing this exercise following sternotomy. A typical exercise session was carried out with a physiotherapist before discharge. Participants were encouraged to maintain their daily activities and continued the usual outpatient clinical care, including medical treatment. They were free to consult a community-based physiotherapist if needed.

This study was conducted within a fixed budget as part of routine care. Physiotherapists ensured in-hospital screening and Vivifrail education, while follow-up visits were carried out in outpatient clinics.

### Outcomes

The primary outcome was to assess feasibility based on the following predefined success criteria:

1. recruitment of at least 70% of the target sample (*n* = 40) within a 12-month recruitment period;
2. retention of at least 70% of participants at W6, defined as completion of the W6 assessment, as previous evidence suggests that the greatest improvements in physical performance typically occur within the first 6 weeks of CR (28);
3. explorationof whether physical performance at W6 in participants adherent to the intervention was not meaningfully worse than that observed in less adherent participants, in order to rule out potential detrimental effects associated with the intervention.

For adherence analyses, participants were categorized post hoc into 2 groups based on programme completion rate: an adherent group (completion rate ≥ 55%) and a non-adherent group (< 55%). This threshold was selected based on previous literature reporting lower adherence rates in community- and home-based exercise programmes among older adults (29,30) compared with higher adherence observed in facility-based CR programmes (31). Given the pilot nature of the study, these analyses were considered exploratory and were not powered to draw a definitive conclusion.

Secondary outcomes included: (*i*) programme completion rate, defined as the proportion of prescribed exercise sessions completed and recorded in the Vivifrail booklet; and (*ii*) changes in physical performance between baseline and W6. Physical performance was assessed using the SPPB score, TUG, and handgrip strength. An improvement of at least 1 point on the SPPB was considered clinically meaningful (32, 33).

### Statistical analysis

Descriptive statistics were used to summarize baseline characteristics. Depending on normality (tested using the Shapiro–Wilk test), continuous variables were compared using Student’s *t*-test or a non-parametric equivalent. To evaluate the effect of the intervention over time, repeated analysis of variance (ANOVA) or paired *t*-tests were performed. To account for confounding factors, mixed linear models were generated with the participant’s identity as random effect. A two-sided *p*-value < 0.05 was considered statistically significant. Analyses were conducted using R software, version 4.5.1 (R Foundation for Statistical Computing, Vienna, Austria).

## RESULTS

### Participants

Patient characteristics are presented in Table I. Thirty participants were men (79%), with a mean age of 79 (3.2) years (range 75–86). The types of surgery performed included mitral or aortic valve replacement (MVR or AVR) in 55% of cases and coronary artery bypass grafting (CABG) in 50%. The characteristics were consistent between the group that underwent a single surgical procedure and the group that underwent multiple procedures.

**Table I:** Characteristics of subjects at baseline

Characteristics	All population (*n* = 38) mean (± SD) or *n* (%)	Patient that underwent >1 type of surgery (*n* = 12)
Sociodemographic data		
Age, years, mean (SD)	79 (± 4)	78 (± 2.9)
Men, *n* (%)	30 (79%)	10 (83%)
BMI, kg/m^2^, mean (SD)	25 (± 3.5)	25.5 (± 3.8)
Vivifrail status at baseline, *n* (%)		
A = severe limitation/dependent or disabled	3 (8)	1 (8)
B/B+ = moderate limitation/frail	16 (42)	5 (42)
C/C+ = mild limitation/prefrail	19 (50)	6 (50)
Type of surgery, *n* (%)		
Coronary artery bypass grafting	17 (45)	7 (58)
Aortic valve replacement	16 (42)	6 (50)
Mitral valve replacement	7 (18)	4 (33)
Bentall	4 (10)	2 (17)
Maze	2 (5)	2 (17)
Transcatheter aortic valve implantation	1 (3)	0 (0)
Myxoma exeresis (left atrium)	1 (3)	0 (0)
Community based physiotherapy, *n* (%)	19 (57)	8 (67)

### Recruitment and retention

**Fig. 1:**
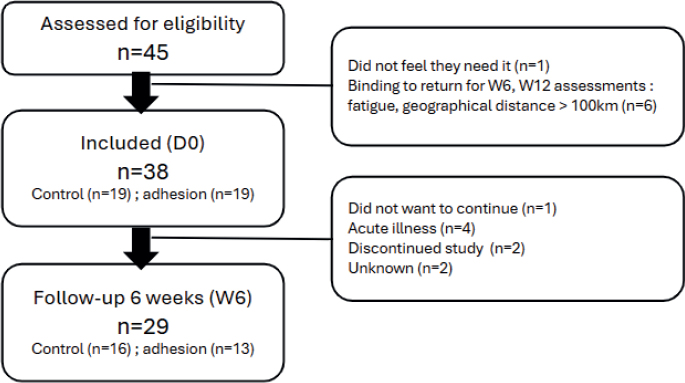
Study flow diagram.

Among 45 eligible patients, 38 (84%) agreed to participate in the programme. Of these, 29 (76%) returned for assessment at W6. The main reasons for non-adherence to the study protocol were fatigue and long distance from home to Nantes University Hospital (sometimes >100 km) (Study flow diagram, Fig. 1). Most patients who missed the W6 visit did so due to an acute illness or because they had been referred to a rehabilitation centre in the meantime.

### Completion rate

A total of 30 exercise sessions were prescribed during the first 6-week intervention period. According to the Vivifrail logbook, the mean completion rate was 53.9%. Of the 38 participants, 11 completed 90% or more; 2 completed 80% or more; 6 completed 60% or more; 2 completed 30%; and 11 completed 20% or less. Data were not reported for 6 participants: 2 discontinued the study (departure to a rehabilitation centre), 1e did not want to continue, and 3 experienced acute medical events (influenza-like illness and stroke).

### Changes in physical performance

**Fig. 2:**
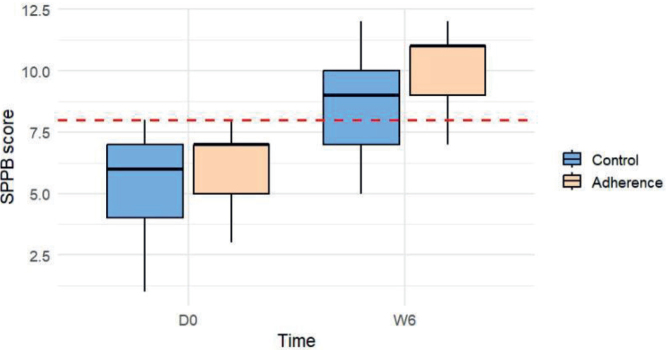
Changes in frailty between groups and over time. Data are median and IQR. Red dashed line represents the cutoff value for frailty (SPPB = 9).^,^ No statistically significant difference between groups.

Most of the improvement occurred within the first 6 weeks (Table II). Participants showed a statistically significant improvement in SPPB score between D0 5.5 (2) and W6 8.6 (2.3), *p*-value for paired *t*-test < 0.001. In a mixed linear model including adherence group, surgery type, sex, and age, changes in SPPB score remained significant over time (adjusted change 2.96, test statistic *t*-value 5.46, *p*-value < 0.001). However, we showed no significant interaction between adherence group and time, with a *p*-value of 0.37 showing no differential improvement between groups over time. Adjusted SPPB progressions for each subgroup are presented in Fig. 2 and Table III.

**Table II:** Changes in physical performance over time

Item	D0 (*n* = 38)	W6 (*n* = 29)
SPPB	5.47 (1.98)	8.62 (2.26)
Balance score	1.00 [1.00–2.00]	3.00 [2.00–4.00]
Walking score	3.00 [2.00–4.00]	4.00 [3.00–4.00]
STS score	1.00 [0.00–1.00]	2.00 [1.00–2.00]
Walking time (s)	5.97 (2.05)	4.58 (1.44)
STS speed (s)	31.17 [18.00–60.00]	15.52 [14.53–19.02]
TUG	17.53 (6.69)	13.32 (4.16)
Handgrip	23.53 (7.80)	24.77 (6.13)

Data are means (standard deviation) or median [IQR]. *Significant difference over time between D0–W6 (*p* < 0.05).

**Table III:** Changes in physical performance over time, depending on completion rate reported in Vivifrail logbook

	Day 0		Week 6	
	Non adherent (*n* = 18)	Adherent (*n* = 20)	Non adherent (*n* = 12)	Adherent (*n* = 17)
SPPB	6.00 [4.00–7.00]	7.00 [5.00–7.00]	9.00 [7.00–10.00]	11.00 [9.00–11.00]
Balance score	1.00 [1.00–2.00]	2.00 [2.00–2.50]	3.00 [2.00–4.00]	4.00 [3.00–4.00]
Walking score	3.00 [2.00–4.00]	3.00 [3.00–4.00]	4.00 [3.00–4.00]	4.00 [4.00–4.00]
STS score	1.00 [0.00–1.00]	1.00 [1.00–1.00]	2.00 [1.00–2.00]	3.00 [2.00–4.00]
Walking time (s)	5.93 [4.39–6.86]	5.29 [4.63–5.85]	4.31 [3.60–5.40]	3.20 [2.80–4.04]
STS speed (s)	31.17 [18.00–60.00]	25.42 [17.61–39.50]	15.52 [14.53–19.02]	13.52 [11.09–16.35]
TUG	19.2 (8.29)	15.8 (4.15)	13.32 (4.16)	11.12 (2.90)
Handgrip	23.53 (7.80)	24.96 (7.69)	24.77 (6.13)	29.59 (9.75)

Data are means (standard deviation) or median [IQR]. No significant difference between groups. SPPB = short performance battery test, STS = sit-to-stand, TUG = Timed Up and Go.

### Changes in TUG, handgrip, and walking speed

At baseline (D0), both the adherence and non-adherence groups were similar in size and performance, confirming initial homogeneity.

All analysed parameters showed statistically significant improvement between D0 and W6:

- TUG time (s): D0 = 17.5 (6.7); W6 = 13.3 (4.2), *p* < 0.001.
- Time for walking 4 m (s): D0 = 6 (2); W6 = 4.6 (1.4), *p* < 0.001.
- Handgrip strength (kg): D0 = 23.5 (7.8); W6 = 24.8 (6.1), *p* < 0.001.

Table III summarizes the progression of these 3 outcomes across all time points for adherent and non-adherent participants.

**Fig. 3:**
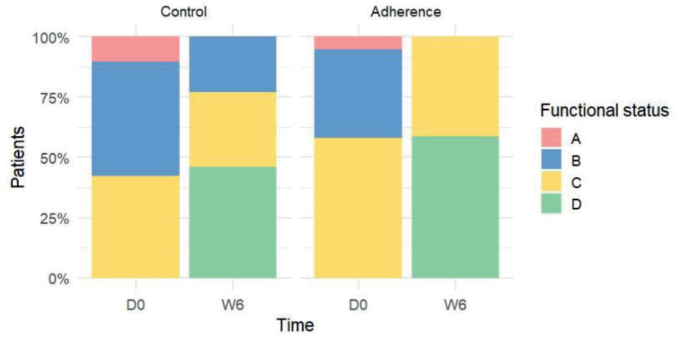
Changes in the functional status according to the Vivifrail classification (http://vivifrail.com/resources/). Frailty is considered reversed when upgrading from A or B to C or D levels.

Although between-group trajectory differences were not statistically significant (see Table III), the adherence group was no longer classified as frail at W6 with a median SPPB score of 11 [9–11] (Fig. 2 and Fig. 3).

## DISCUSSION

This study highlights the potential benefits of Vivifrail as a structured, home-based physical activity programme among frail older adults following cardiac surgery. It proved to be feasible in this population. Clinically meaningful improvements were observed in physical performance and mobility during the first 6 weeks of the programme.

Functional ability (i.e., the capacity to perform activities that individuals value) depends on the interaction between intrinsic capacity (i.e., physical and mental capabilities), environmental factors, and their dynamic interplay (34, 35). Even short periods of inactivity (e.g., during hospitalization or postoperative recovery) can lead to rapid declines in muscle mass and functional capacity (36).

In our population, the pattern of improvement is consistent with the current literature, which shows that recovery after cardiac surgery follows a gradual trajectory over time. The first 3 months represent the period of greatest recovery (37), with the most substantial gains in physical performance occurring within the first 6 weeks, depending on the domains assessed (e.g., 1 to 3 weeks: anxiety, depression, mental quality of life, dyspnoea; 4 to 6 weeks: 6-minute walk test, physical quality of life) (28).

The mean completion rate in our cohort (≈54%) aligns with previous reports for home-based rehabilitation programmes such as the New Zealand Otago (42%) and LiFE (47%) trials (29, 30). Nevertheless, it remains lower than rates reported by the Vivifrail research group: 79% in the first month to 68% over the following 2 months (21).

Several factors may explain this discrepancy. Unlike the original Vivifrail studies, our protocol did not include scheduled telephone reinforcement or systematic caregiver involvement. Instead, participants were solely responsible for monitoring their programme, with in-person follow-ups only at weeks 6 and 12. This approach was chosen based on our cohort’s relatively favourable functional profile, characterized by good physical reserves, rapid functional recovery, and limited limitations on activities (16). Some participants may have perceived less need for ongoing exercise once they regained independence.

Moreover, recent evidence suggests that adherence is not necessarily associated with intervention efficacy (38). Our findings align with this, as we observed no difference between adherence groups, and no interaction between changes in SPPB scores and programme completion. Qualitative feedback further revealed that many participants resumed their usual activities early in recovery, effectively substituting the Vivifrail programme with daily physical tasks (39).

These findings underscore the importance of addressing social and motivational factors influencing adherence. Social isolation, lack of support, or limited feedback mechanisms likely contributed to early discontinuation. Future interventions should consider involving caregivers and offering remote supervision to sustain engagement at home. Collaboration with community-based physiotherapists who can provide support and follow-up care outside of the hospital also represents a promising strategy to consolidate continuity of care. In addition, promoting physical activity as part of everyday life, rather than relying solely on a structured programme, may help ensure that gains made during exercise sessions transfer to activities of daily living and are sustained over time.

Regarding the specific effectiveness of the Vivifrailprogramme, time seemed to be more beneficial than the programmeitself. Indeed, our results did not show any statistical difference between adherence groups. Moreover, there was no significant interaction between time and adherence group in mixed linear models, suggesting no differential effect of the programme’s adherence on functional recoveries. This contradicts previous evidence, which supported the effectiveness of multimodal training in maintaining functional independence (6, 8, 9). Nevertheless, we still observed a clinical trend in favour of the adherent group, with fewer participants classified as frail as soon as W6.

These results may be explained by several factors described earlier: (*i*) a lower completion rate than in previous Vivifrail interventions; (*ii*) specific characteristics of our cohort (good physical reserves, rapid functional recovery, and limited limitations on activities); (*iii*) limited sample size. As our primary objective was not to determine the specific factors responsible for improvements in physical capacities, additional community-based physiotherapy was not considered a confounding factor requiring specific adjustment. Qualitative analyses suggested heterogeneous interactions between physiotherapy and engagement with the Vivifrail programme, with some participants relying mainly on physiotherapy whilst others perceived the 2 approaches as complementary (39).

### Strengths and limitations

This study is, to our knowledge, the first to evaluate the Vivifrail programme in older adults following cardiac surgery. The cohort was representative of this population, particularly in terms of surgical procedures (40). Additionally, most eligible patients were enrolled (84%), supporting the internal validity of our observations.

However, several limitations must be acknowledged. The small sample size and high attrition rate substantially reduced statistical power. Our 55% adherence threshold may seem arbitrary. The cutoff point was established based on previous studies on home-based rehabilitation programmes for older patients. However, other adherence cutoff points were also tested (60%, 65%, 70%) and did not change the overall results or conclusions. Participants who dropped out tended to have recovered more rapidly and reached acceptable functional levels by W6, suggesting a potential selection bias toward those with lower baseline function remaining at follow-up. This contrasts with previous literature, as it is described that patients who tended to drop out were older and had worse exercise capacities at baseline (13). Instead, all of our dropouts started the programme with a C booklet indicating good initial function. Thus, the current Vivifrail programme seems to meet the needs of old adults with limited capacity following cardiac surgery. However, adjustments may be needed in terms of how it is applied or monitored for patients with good initial function.

### Conclusion

Home-based multimodal rehabilitation using the Vivifrail programme may be a suitable approach for older adults with limited functional capacity after cardiac surgery, although adaptations may be needed for those with higher baseline function. Future interventions should consider caregiver involvement, remote supervision, and integration of physical activity in daily life to support sustained engagement and long-term functional gains. The Vivifrail programme, aligned with ICOPE principles, should be considered in post-cardiac surgery rehabilitation strategies for frail older adults who cannot be referred to specialized rehabilitation centres.

## References

[1] Nakano M, Nomura Y, Suffredini G, Bush B, Tian J, Yamaguchi A et al. Functional outcomes of frail patients after cardiac surgery: an observational study. Anesth Analg 2020; 130: 1534–1544. 10.1213/ANE.0000000000004786 32384343 PMC7641106

[2] Lin HS, Watts JN, Peel NM, Hubbard RE. Frailty and post-operative outcomes in older surgical patients: a systematic review. BMC Geriatr 2016; 16: 157. 10.1186/s12877-016-0329-8 27580947 PMC5007853

[3] Ijaz N, Buta B, Xue QL, Mohess DT, Bushan A, Tran H, et al. Interventions for frailty among older adults with cardiovascular disease: JACC state-of-the-art review. J Am Coll Cardiol 2022; 79: 482–503. 10.1016/j.jacc.2021.11.029 35115105 PMC8852369

[4] Morley JE, Vellas B, van Kan GA, Anker SD, Bauer JM, Bernabei R, et al. Frailty consensus: a call to action. J Am Med Dir Assoc 2013; 14: 392–7. 10.1016/j.jamda.2013.03.022. 23764209 PMC4084863

[5] Veronese N. Frailty and cardiovascular diseases: research into an elderly population. Vol. 1216. C ha, Switzerland: Springer International Publishing; 2020. 10.1007/978-3-030-33330-0

[6] Brennan TH, Lewis LK, Gordon SJ, Prichard I. Effectiveness of interventions to prevent or reverse pre-frailty and frailty in middle-aged community dwelling adults: a systematic review. Prev Med 2024; 185: 108008. 10.1016/j.ypmed.2024.108008. 38797264

[7] Kolle AT, Lewis KB, Lalonde M, Backman C. Reversing frailty in older adults: a scoping review. BMC Geriatr 2023; 23: 751. 10.1186/s12877-023-04309-y. 37978444 PMC10655301

[8] Anderson L, Oldridge N, Thompson DR, Zwisler AD, Rees K, Martin N, et al. Exercise-based cardiac rehabilitation for coronary heart disease: Cochrane systematic review and meta-analysis. J Am Coll Cardiol 2016; 67: 1–12. 10.1016/j.jacc.2015.10.044 26764059

[9] Karnon J, Afzali HHA, Putro GVAA, Thant PW, Dompok A, Cox I, et al. A cost-effectiveness model for frail older persons: development and application to a physiotherapy-based intervention. Appl Health Econ Health Policy 2017; 15: 635–645. 10.1007/s40258-017-0324-z 28349499

[10] Bjarnason-Wehrens B, McGee H, Zwisler AD, Piepoli MF, Benzer W, Schmid JP, et al.; Cardiac Rehabilitation Section European Association of Cardiovascular Prevention and Rehabilitation. Cardiac rehabilitation in Europe: results from the European Cardiac Rehabilitation Inventory Survey. Eur J Cardiovasc Prev Rehabil 2010; 17: 410–18. 10.1097/HJR.0b013e328334f42d 20300001

[11] Kotseva K, De Backer G, De Bacquer D, Rydén L, Hoes A, Grobbee D, et al. Lifestyle and impact on cardiovascular risk factor control in coronary patients across 27 countries: results from the European Society of Cardiology ESC-EORP EUROASPIRE V registry. Eur J Prev Cardiol 2019; 26: 824–835. 10.1177/2047487318825350 30739508

[12] Lutz AH, Forman DE. Cardiac rehabilitation in older adults: apropos yet significantly underutilized. Prog Cardiovasc Dis 2022; 70: 94–101. 10.1016/j.pcad.2022.01.001 35016915 PMC8930627

[13] Brouwers RWM, Houben VJG, Kraal JJ, Spee RF, Kemps HMC. Predictors of cardiac rehabilitation referral, enrolment and completion after acute myocardial infarction: an exploratory study. Neth Heart J 2021; 29: 151–157. 10.1007/s12471-020-01492-0 33030659 PMC7904980

[14] Essery R, Geraghty AW, Kirby S, Yardley L. Predictors of adherence to home-based physical therapies: a systematic review. Disabil Rehabil 2017; 39: 519–534. 10.3109/09638288.2016.1153160 27097761

[15] Clark AM, King-Shier KM, Duncan A, Spaling M, Stone JA, Jaglal S, et al. Factors influencing referral to cardiac rehabilitation and secondary prevention programs: a systematic review. Eur J Prev Cardiol 2013; 20: 692–700. 10.1177/2047487312447846 23847263

[16] Racine G, Huet J, Nicollet J, Roussel JC, Boureau AS. Dépistage d’altération des capacités intrinsèques via l’outil ICOPE à la suite d’une chirurgie cardiaque: implémentation, efficacité de l’intervention et évolution de la qualité de vie. Brest, France: SGOC; 2025.

[17] Thomas RJ, Beatty AL, Beckie TM, Brewer LC, Brown TM, Forman DE. et al. Home-based cardiac rehabilitation: a scientific statement from the American Association of Cardiovascular and Pulmonary Rehabilitation, the American Heart Association, and the American College of Cardiology. Circulation 2019; 140: e69–89. 10.1016/j.jacc.2019.03.008 31082266

[18] McDonagh ST, Dalal H, Moore S, Clark CE, Dean SG, Jolly K, et al. Home-based versus centrebased cardiac rehabilitation. Cochrane Database Syst Rev 27 2023; 10: CD007130. 10.1002/14651858.CD007130.pub5 PMC10604509 37888805

[19] Integrated care for older people: guidelines on community-level interventions to manage declines in intrinsic capacity. Geneva: World Health Organization; 2017. 29608259

[20] Martínez-Velilla N, Casas-Herrero A, Zambom-Ferraresi F, et al. Effect of exercise intervention on functional decline in very elderly patients during acute hospitalization: a randomized clinical trial. JAMA Intern Med 2019; 179: 28. 10.1001/jamainternmed.2018.7586 30419096 PMC6583412

[21] Casas-Herrero Á, Sáez de Asteasu Ml, Antón-Rodrigo I, Sánchez-Sánchez Jl, Montero-Odasso M, Marín-Epelde I, et al. Effects of Vivifrail multicomponent intervention on functional capacity: a multicentre, randomized controlled trial. J Cachexia Sarcopenia Muscle 2022; 13: 884–893. 10.1002/jcsm.12925 35150086 PMC8977963

[22] Aguado Ortego R, Gómez González L, Gómez Rubiano M, Fernández Rodriguez E, Baztán Cortés JJ, Gómez-Pavón J. Beneficios de un programa ambulatorio de ejercicio multicomponente unido a la valoración geriátrica integral para pacientes mayores de la comunidad con fragilidad física [Benefits of an outpatient multicomponent exercise program combined with comprehensive geriatric assessment for elderly community patients with physical frailty]. Rev Esp Geriatr Gerontol 2025; 60: 101625. Spanish. 10.1016/j.regg.2025 39947016

[23] Cruz-Jentoft AJ, Bahat G, Bauer J, Boirie Y, Bruyère O, Cederholm T, et al. Sarcopenia: revised European consensus on definition and diagnosis. Age Ageing 2019; 48: 16–31. https://doi.org/10.1093/ageing/afy169. Erratum in: Age Ageing 2019; 48: 601. https://doi.org/10.1093/ageing/afz046 30312372 10.1093/ageing/afy169 PMC6322506

[24] Perracini MR, Mello M, de Oliveira Máximo R, Bilton TL, Ferriolli E, Lustosa LP, et al. Diagnostic accuracy of the Short Physical Performance Battery for detecting frailty in older people. Phys Ther 2020; 100: 90–98. 10.1093/ptj/pzz154 31612228

[25] Lee L, Patel T, Costa A, Bryce E, Hillier LM, Slonim K, et al. Screening for frailty in primary care: Accuracy of gait speed and hand-grip strength. Can Fam Physician 2017; 63: e51–e57. 28115460 PMC5257239

[26] Ruiz-Ruiz L, Jimenez AR, Garcia-Villamil G, Seco F. Detecting fall risk and frailty in elders with inertial motion sensors: a survey of significant gait parameters. Sensors 2021; 21: 6918. 10.3390/s21206918 34696131 PMC8538337

[27] Izquierdo M. Prescripción de ejercicio físico. El programa Vivifrail como modelo [Multicomponent physical exercise program: Vivifrail]. Nutr Hosp 2019; 36(Spec. No. 2): 50–56. Spanish. 10.20960/nh.02680 31189323

[28] Petersen J, Vettorazzi E, Winter L, Schmied W, Kindermann I, Schäfers HJ. Physical and mental recovery after conventional aortic valve surgery. J Thorac Cardiovasc Surg 2016; 152: 1549–1556.e2. 10.1016/j.jtcvs.2016.07.072 27751583

[29] Campbell AJ, Robertson MC, Gardner MM, Norton RN, Tilyard MW, Buchner DM. Randomised controlled trial of a general practice programme of home based exercise to prevent falls in elderly women. BMJ 1997; 315: 1065–1069. 10.1136/bmj.315.7115.1065 9366737 PMC2127698

[30] Clemson L, Fiatarone Singh MA, Bundy A, Cumming RG, Manollaras K, O’Loughlin P, et al. Integration of balance and strength training into daily life activity to reduce rate of falls in older people (the LiFE study): randomised parallel trial. BMJ 2012; 345: e4547. 10.1136/bmj.e4547. 22872695 PMC3413733

[31] Jansson AK, Lubans DR, Duncan MJ, Smith JJ, Bauman A, Attia J, et al. Increasing participation in resistance training using outdoor gyms: a study protocol for the ecofit type III hybrid effectiveness implementation trial. Contemp Clin Trials Commun 2024; 41: 101358. 10.1016/j.conctc.2024.101358 39280786 PMC11399599

[32] Perera S, Mody SH, Woodman RC, Studenski SA. Meaningful change and responsiveness in common physical performance measures in older adults. J Am Geriatr Soc 2006; 54: 743–749. 10.1111/j.1532-5415.2006.00701.x. 16696738

[33] Kameniar K, Mackintosh S, Van Kessel G, Kumar S. The psychometric properties of the short physical performance battery to assess physical performance in older adults: a systematic review. J Geriatr Phys Ther 2022; 47 :43–54. 10.1519/jpt.0000000000000337 35442231

[34] Cesari M, Araujo de Carvalho I, Amuthavalli Thiyagarajan J, Cooper C, Martin FC, Reginster JY, et al. Evidence for the domains supporting the construct of intrinsic capacity. J Gerontol A Biol Sci Med Sci 2018; 73: 1653–1660. 10.1093/gerona/gly011 29408961

[35] Yu R, Leung J, Leung G, Woo J. Towards healthy ageing: using the concept of intrinsic capacity in frailty prevention. J Nutr Health Aging 2022; 26: 30–36. 10.1007/s12603-021-1715-2 35067700 PMC12275532

[36] Shur NF, Creedon L, Skirrow S, Atherton PJ, MacDonald IA, Lund J, et al. Age-related changes in muscle architecture and metabolism in humans: the likely contribution of physical inactivity to age-related functional decline. Ageing Res Rev 2021; 68: 101344. 10.1016/j.arr.2021.101344 33872778 PMC8140403

[37] Coelho PNMP, Miranda LMRPC, Barros PMP, Fragata JIG. Quality of life after elective cardiac surgery in elderly patients. Interact Cardiovasc Thorac Surg 2019; 28: 199–205. 10.1093/icvts/ivy235 30085061

[38] Wu S, Nan J, Chang J, Jiang D, Cao Z, Zhou S, et al. Adherence to exercise intervention for community-dwelling older adults with sarcopenia: a systematic review and meta-analysis. Age Ageing 2025; 54(4): afaf094. 10.1093/ageing/afaf094 40253683

[39] Komonski A, Meunier C, Boureau AS. Factors influencing the implementation of a home-based rehabilitation program in frail older patients after cardiac surgery: a qualitative study using reflexive thematic analysis. BMC Sports Sci Med Rehabil 2026. Online ahead of print. 10.1186/s13102-026-01696-8 PMC13227708 42015305

[40] Lee S, Chang BC, Yoo KJ. Surgical management of coexisting coronary artery and valvular heart disease. Yonsei Med J 2010; 51: 326–331. 10.3349/ymj.2010.51.3.326 20376883 PMC2852786

